# Right colon interposition in benign esophageal stricture and gastric atrophy: technical nuances and functional outcomes from a complex case

**DOI:** 10.1093/jscr/rjaf757

**Published:** 2025-09-25

**Authors:** Santiago Muñoz-Palomeque, Álvaro Morillo Cox, Tatiana Fernández, Mauricio Morillo

**Affiliations:** Department of Surgery, Division of General Surgery, Hospital Metropolitano, Quito 170508, Ecuador; Faculty of Medical, Health and Life Sciences, Universidad Internacional del Ecuador, Quito 170411, Ecuador; Department of Surgery, Hospital de Especialidades Carlos Andrade Marín, Quito 170402, Ecuador; Faculty of Medical, Health and Life Sciences, Universidad Internacional del Ecuador, Quito 170411, Ecuador; Department of Surgery, Hospital General del Sur de Quito, Quito 170401, Ecuador; Department of Surgery, Hospital General del Sur de Quito, Quito 170401, Ecuador; Faculty of Medicine, Universidad de las Américas, Quito 170513, Ecuador; Department of Surgery, Hospital de Especialidades Carlos Andrade Marín, Quito 170402, Ecuador

**Keywords:** colonic interposition, colon surgery specialty, esophageal reconstruction, esophageal stenosis, esophagectomy

## Abstract

We present the case of a 43-year-old woman with complete esophageal and gastric stenosis after caustic ingestion. Following initial emergency surgery for gastric perforation, she remained dependent on a jejunostomy for over a year, developing severe malnutrition, dysphagia, and psychological decline. Definitive reconstruction was achieved with a one-stage subtotal esophagectomy and total gastrectomy. A right colon interposition graft, routed through the posterior mediastinum, was used after confirming adequate perfusion despite the absence of the right colic artery. The procedure included a cervical esophago-colonic anastomosis, Roux-en-Y colojejunal anastomosis, ileotransverse bypass, and incidental cholecystectomy and appendectomy. Postoperative recovery was uneventful, with the patient resuming soft oral intake, gaining weight, and showing marked nutritional and functional improvement. This case underscores the value of right colon interposition when the stomach is unavailable, highlighting the need for tailored conduit choice, precise vascular assessment, and multidisciplinary perioperative care in complex benign esophageal disease.

## Introduction

Caustic ingestion of strong acids or alkalis can result in severe damage to the upper gastrointestinal tract, particularly the esophagus and stomach. The extent of injury depends on the nature of the substance and duration of exposure. Alkali agents typically induce liquefactive necrosis with deep transmural injury, whereas acids cause coagulative necrosis with eschar formation that may limit deeper penetration [1]. Acute complications include perforation, fistula formation, and peritonitis or mediastinitis, with a mortality rate of up to 20% in severe cases [2]. Survivors frequently develop long-term sequelae such as esophageal strictures, gastric outlet obstruction, and increased risk of esophageal carcinoma [1]. Initial management is conservative, focused on limiting acute damage and preventing stricture formation, but after the acute phase has resolved, if patients develop long-segment strictures refractory to endoscopic dilatation—or complete obstruction—surgical intervention becomes necessary to restore alimentary continuity [3].

Colon interposition, first described in the early 20th century and popularized by surgeons such as Richardsons and Belsey, is a well-established technique for esophageal reconstruction [4, 5]. It is indicated in cases of trauma, caustic injury, malignancy, or failed prior reconstructions where gastric conduits are unsuitable or unavailable [6–9]. The colon offers reliable length, acid resistance, and a robust marginal arterial arcade (Drummond’s marginal artery) [10]. While the left colon is most commonly used, right colon interposition is a valid alternative depending on vascular anatomy and surgical preference [6, 10].

This report presents a case of severe caustic-induced esophageal and gastric injury managed with right colon interposition, highlighting the pathophysiological basis, surgical indications, and key technical considerations in this complex reconstructive approach.

## Case report

A 44-year-old woman with a history of caustic ingestion during a suicide attempt 18 months prior initially presented with Zargar grade IIIB esophagitis and erosive gastropathy, complicated by proximal gastric perforation and secondary peritonitis. Emergency surgery was performed, including exploratory laparotomy, primary gastric repair, Dor fundoplication, peritoneal lavage, and creation of a Witzel-type feeding jejunostomy.

**Figure 1:**
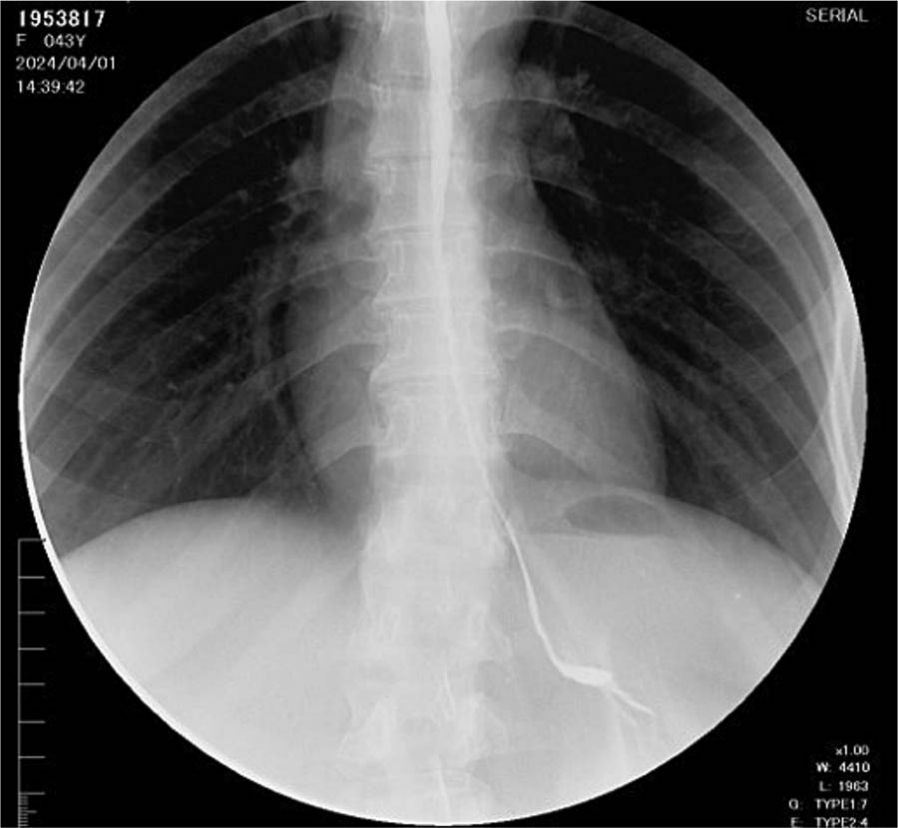
Esophagogram with esophageal stricture.

During follow-up, the patient developed complete esophageal stricture (Fig. 1) with absolute oral intolerance, leading to prolonged dependence on jejunal feeding. This resulted in progressive malnutrition, generalized sarcopenia, and a body mass index of 15 kg/m^2^. Given failure of endoscopic management, definitive esophageal reconstruction was indicated. Preoperative outpatient optimization included high-calorie enteral supplementation and monitoring of albumin, prealbumin, and total protein levels. Psychiatric evaluation confirmed emotional stability and adherence to pharmacologic treatment, clearing her for surgery.

A midline laparotomy was performed with extensive adhesiolysis. A total gastrectomy and near-total esophagectomy were completed using the Orringer technique, incorporating a left cervical incision along the anterior border of the sternocleidomastoid muscle. The previously placed jejunostomy was removed. The stomach was atrophic and nonviable as a conduit.

**Figure 2:**
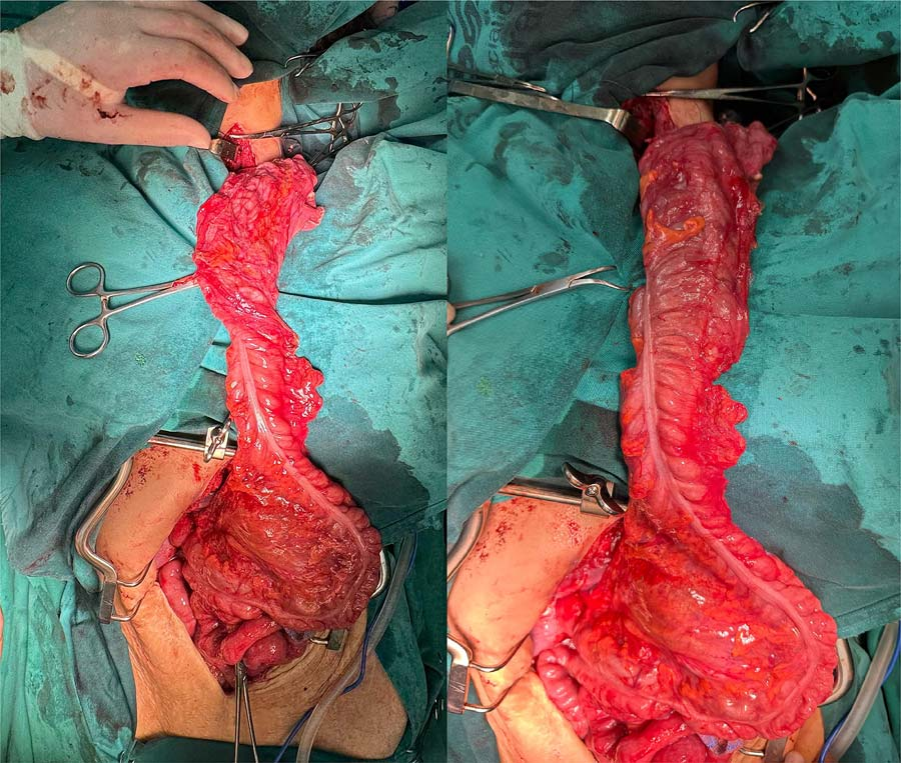
Right colonic interposition after Cattel-Braasch maneuver.

**Figure 3:**
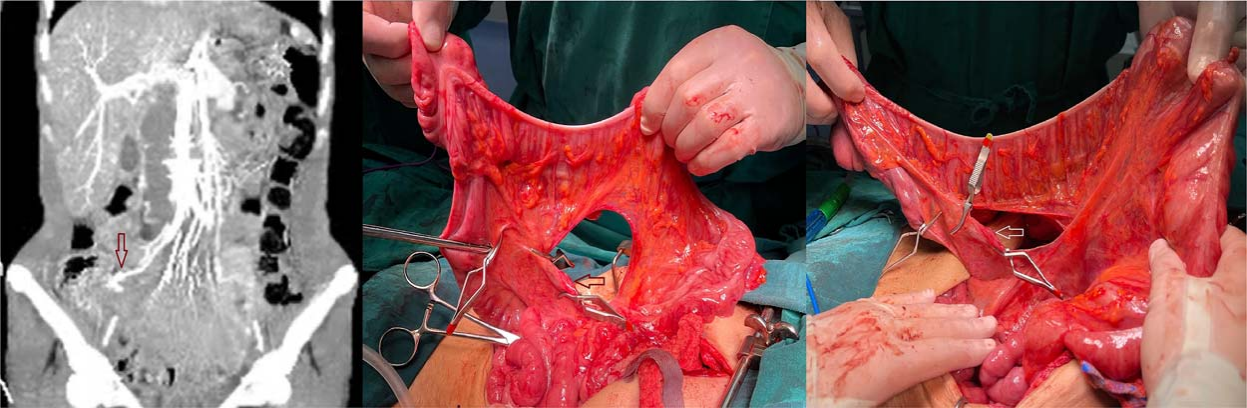
Confirmation of collateral vascular flow in the right colon (arrow shows ileocolic artery).

The right colon was mobilized using a standard Cattell–Braasch maneuver. Intraoperative assessment revealed the absence of the right colic artery; perfusion relied on the ileocolic artery and the right branch of the middle colic artery via a well-formed marginal artery of Drummond (Fig. 2). Temporary occlusion of the ileocolic vessels confirmed adequate collateral flow (Fig. 3). The terminal ileum was transected ~3 cm proximal to the ileocecal valve, and an incidental appendectomy was performed.

**Figure 4:**
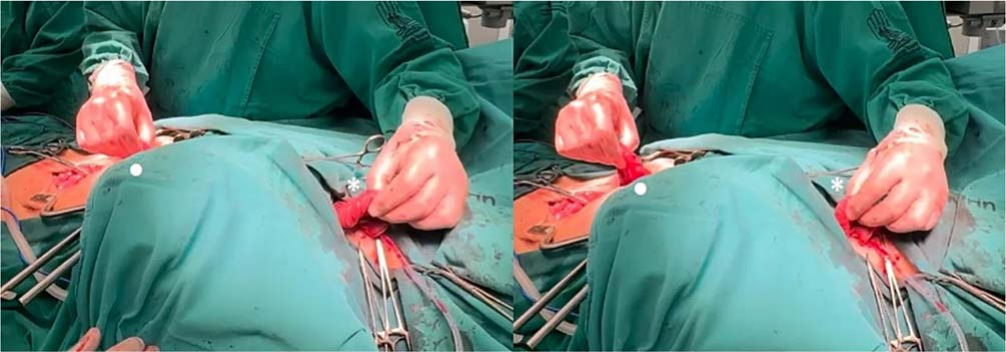
Positioning of the right colon in the posterior mediastinum, prior to the formation of the anastomoses (• abdominal/distal end of the right colon, ^*^ cervical/proximal end of the right colon).

**Figure 5:**
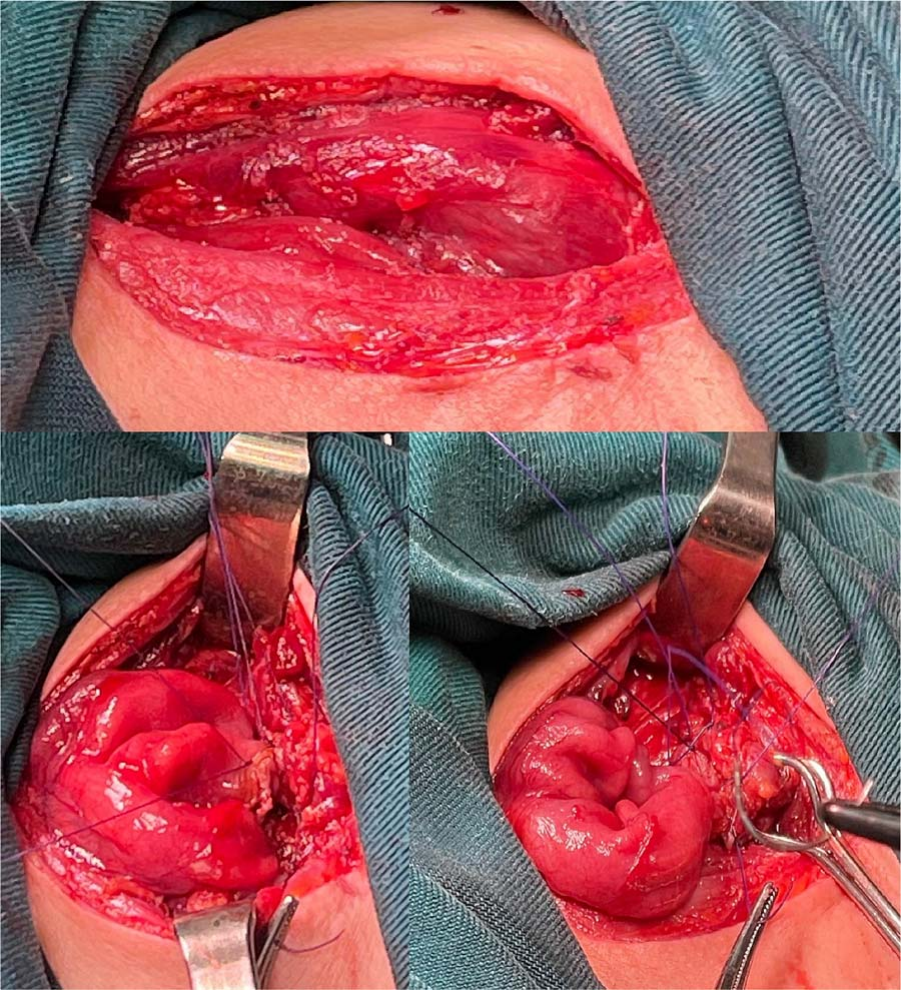
Esophagocolonic anastomosis below the hypopharynx after Orringer’s approach technique.

The prepared right colonic segment was transposed through the posterior mediastinum using the ‘shoe-shiner’ technique to avoid torsion and preserve orientation (Fig. 4). A hand-sewn, two-layer, isoperistaltic esophago-colonic anastomosis was performed in the cervical region using interrupted 4–0 polyglactin sutures for the mucosal layer and running seromuscular sutures for the outer layer (Fig. 5).

**Figure 6:**
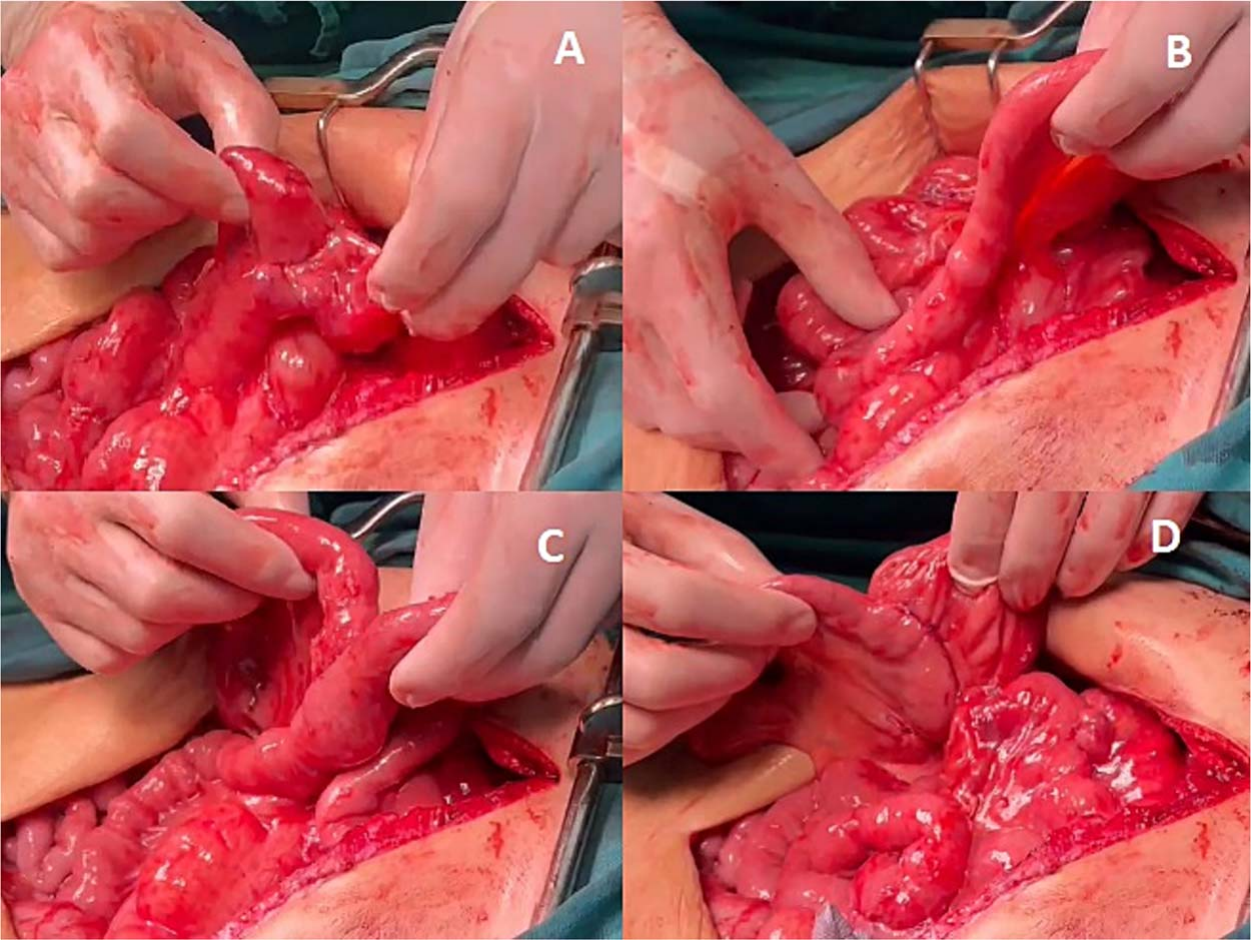
Abdominal anastomoses. (A) Colo-jejunal anastomosis, (B) alimentary loop, (C) Roux-en-Y anastomosis, (D) ileo-transverse anastomosis.

Alimentary tract continuity was re-established with a mechanical, side-to-side, isoperistaltic Roux-en-Y colojejunal anastomosis and a side-to-side ileotransverse anastomosis (Fig. 6). Two closed-suction drains were placed: one adjacent to the cervical anastomosis and another via the esophageal hiatus into the posterior mediastinum. Total operative time was 320 minutes, and estimated blood loss was 1000 ml.

The patient was admitted to the intensive care unit for 48 hours, requiring low-dose vasopressors but no mechanical ventilation. Parenteral nutrition began on postoperative day 1 (POD1). On POD2, clear liquids were trialed but limited by dysphagia; flexible endoscopy carried by the otolaryngology team ruled out obstruction. A semisolid diet was started under swallowing therapy and was well tolerated. Drains were removed on POD7, and the patient was discharged on a soft diet with good bowel function and no signs of infection or anastomotic leak.

At first follow-up, she showed weight gain, adherence to nutritional goals, and stable psychiatric status under continued outpatient care.

## Discussion

Colonic interposition remains one of the most technically demanding operations in gastrointestinal surgery with a significant learning curve and a high risk of early complications such as graft necrosis or anastomotic leaks and late complications such as stricture, redundancy, and dysmotility [4, 6, 9, 11–13].

However, colonic interposition remains a technically demanding yet valuable option for esophageal reconstruction in patients with benign pathology where the stomach is unavailable due to caustic damage or prior surgery. In this context, the right colon, based on the middle colic artery, offers specific advantages over the left colon, particularly in terms of length, reach, and isoperistaltic alignment [4, 5, 7]. Although the left colon has traditionally been preferred due to consistent vascular anatomy, recent literature supports the right colon as a reliable and functionally effective alternative when vascular integrity is confirmed [5, 9].

The vascular anatomy relevant to colon interposition enhances the marginal artery of Drummond, first described in 1914, which provides the critical anastomotic channel linking the ileocolic, right colic, middle colic, and left colic arteries [14]. While often depicted as a continuous arcade, anatomical studies have shown that the marginal artery can be incomplete or tenuous in some patients, particularly at Griffith’s point (splenic flexure) and Sudeck’s point (sigmoid-rectal junction) [10].

In our case, the absence of the right colic artery and dominant supply via the ileocolic and middle colic arteries was confirmed intraoperatively, with a robust marginal artery of Drummond ensuring perfusion. This eliminated the need for supercharging, which may be reserved for questionable vascularization or very long conduits [7, 12, 15]. The posterior mediastinal route was selected before retrosternal and subcutaneous routes due to prior resection of the native esophagus and stomach, providing a natural and anatomical path [9]. This route minimizes redundancy, allows for smoother conduit passage, and reduces the risk of compression depending on the patient’s anatomy [5, 11, 15].

Surgical precision—including manual two-layer esophago-colonic anastomosis, mechanical colojejunal anastomosis, and the ‘shoe shiner’ maneuver to avoid torsion—was critical. The favorable postoperative course, absence of leak or ischemia, and rapid nutritional recovery underscore the effectiveness of the meticulous technique and individualized conduit planning. Nutritional optimization pre- and postoperatively also played a pivotal role in reducing complications and supporting anastomotic healing.

## Conclusions

This case highlights the successful use of right colon interposition for esophageal reconstruction in a patient with long-segment caustic esophageal and gastric strictures, where conventional options such as gastric pull-up were not feasible. The patient demonstrated excellent postoperative outcomes, including restoration of oral intake, significant nutritional recovery, and stability of preexisting psychiatric comorbidities, all of which contributed to a favorable quality of life after surgery.

The complexity of this case underscores the critical importance of multidisciplinary evaluation, involving surgical, nutritional, psychiatric, and anesthetic teams, in managing patients with severe caustic injuries. Equally essential is the role of experienced surgical teams familiar with advanced reconstructive techniques and the nuances of colonic vascular anatomy.

Right colon interposition via the posterior mediastinum proved to be a safe and effective solution, provided that vascular integrity is confirmed and meticulous surgical technique is employed. This experience reinforces the notion that with proper case selection and comprehensive perioperative care, even the most challenging benign esophageal pathologies can be managed with excellent functional results.

## Data Availability

All data presented are available from the corresponding author.
